# Engineering Cobalt-Based Bimetallic Compounds via NH_4_F‑Directed ZIF67 Transformation for Battery–Supercapacitor Hybrids with Enhanced Energy Storage Performance

**DOI:** 10.1021/acsomega.5c04906

**Published:** 2025-08-22

**Authors:** Tsai-Mu Cheng, Yi-An Lu, Pin-Chun Lee, Chutima Kongvarhodom, Sadang Husain, Sibidou Yougbaré, Hung-Ming Chen, Lu-Yin Lin

**Affiliations:** a Graduate Institute for Translational Medicine, College of Medical Science and Technology, Taipei Medical University, Taipei 11031, Taiwan; b Taipei Heart Institute, Taipei Medical University, Taipei 11031, Taiwan; c Cardiovascular Research Center, Taipei Medical University Hospital, Taipei 11031, Taiwan; d Department of Chemical Engineering and Biotechnology, 34877 National Taipei University of Technology, Taipei 10608, Taiwan; e Department of Chemical Engineering, 65128 King Mongkut’s University of Technology Thonburi, 126 Pracha-u-thit, Toong-kru, Bangkok 10140, Thailand; f Department of Physics, Faculty of Mathematics and Natural Science, 175513 Lambung Mangkurat University, Banjarmasin 70124, Indonesia; g 307955 Institut de Recherche en Sciences de la Santé (IRSS-DRCO)/Nanoro, 03 BP 7192, Ouagadougou 7192-03, Burkina Faso; h Gingen Technology Co., Ltd., Rm. 7, 10 F., No.189, Sec. 2, Keelung Rd., Xinyi Dist., Taipei 11054, Taiwan

## Abstract

Cobalt-based compounds have attracted considerable attention as electroactive materials for energy storage owing to their high theoretical capacitance and cost-effectiveness. Zeolitic imidazolate framework-67 (ZIF67) is a cobalt-containing metal–organic framework that features a high surface area and a tunable porous architecture, but inherently low conductivity limits its electrochemical performance. To address this issue, structure-directing agents (SDAs) have been employed to enhance the surface characteristics and energy storage behavior of ZIF67 derivatives. In particular, the redox activity of battery-type electrodes is largely governed by the nature of the metal species involved. In this study, a series of cobalt-based bimetallic compounds incorporating Ni, Cu, Al, Zn, and Mn are synthesized using NH_4_F as the SDA in 2-methylimidazole medium. Morphology and composition of the resulting materials are strongly dependent on the secondary metal species. The cobalt–nickel (CoNi) electrode achieves the highest specific capacitance (*C*_F_) of 997.3 F/g at 20 mV/s, attributed to the synergistic redox behavior of cobalt and nickel. The contributions from both diffusion-controlled and surface-capacitive processes are also quantitatively assessed. A BSH assembled using the CoNi and carbon electrodes achieves a maximum energy density of 9.2 Wh/kg at 375 W/kg, along with a *C*_F_ retention of 83.1% and a Coulombic efficiency of 94.2% after 10,000 cycles.

## Graphical abstract

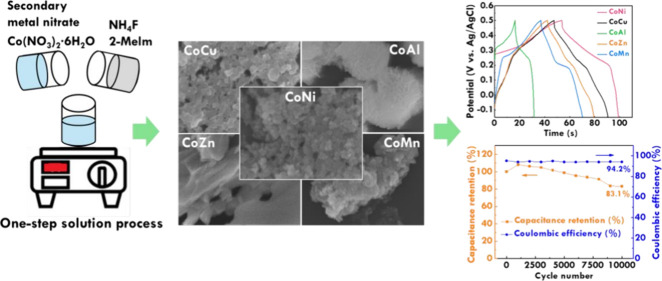

## 1 Introduction

Electrochemical energy storage devices have been widely employed to address global energy and environmental challenges, including air and water pollution and intermittent energy supply.^1–9^ Among these issues, energy storage plays a pivotal role in stabilizing power grids and supporting renewable energy integration, particularly by providing electricity during peak demand periods.^10^ The effectiveness of such devices largely depends on their energy and power densities.^11^ Two major types of storage systems, batteries and supercapacitors, have been developed to optimize these parameters. Batteries store energy via redox reactions, enabling high energy density, whereas supercapacitors rely on electric double-layer capacitance (EDLC), which supports rapid charge/discharge and high power density.^12–14^ Recently, battery–supercapacitor hybrids (BSHs) that integrate battery-type and capacitor-type electrodes have attracted increasing attention due to their combined advantages.^15–19^ In such systems, the battery-type positive electrode determines the energy density, while the capacitor-type negative electrode (commonly carbon-based) ensures fast kinetics. Since carbon materials are already well-established, research has shifted toward developing advanced electroactive materials for the battery-type electrode to further improve overall BSH performance.^20^

The battery-type electrode consists of a current collector and an electroactive material, and the latter is critical in defining the energy storage capacity. Redox reactions occurring at the electrode–electrolyte interface govern the charge storage process,^21–23^ and their efficiency is strongly influenced by the intrinsic properties of the active material, including metal species, chemical composition, crystal structure, surface area, and porosity.^24^ Optimizing these characteristics is essential for enhancing the performance of BSHs. Structure-directing agents (SDAs) are often employed to regulate material morphology and facilitate the formation of electrochemically favorable structures.^18,25–27^ Metal–organic frameworks (MOFs), especially zeolitic imidazolate frameworks (ZIFs), are promising precursors for electroactive materials due to their high surface areas and tunable architectures.^28–30^ To enhance their performance, SDAs such as NH_4_F have been used to tailor the morphology and composition of ZIF67, a cobalt-based MOF.^31–36^ Compared to conventional postsynthesis treatments (e.g., carbonization or sulfurization), NH_4_F-induced transformation offers superior control and improved electrochemical activity. In this context, NH_4_^+^ serves as a pH regulator and potential charge attractor, while the highly electronegative F^–^ ions can promote the conductivity and influence phase formation. Furthermore, metal ions play a central role in redox behavior and energy storage efficiency.^37–41^ For example, Yu et al. synthesized MOF derivatives from six metal species (Al, Mn, Co, Ni, Cu, and Zn) using NH_4_BF_4_ and NH_4_HF_2_ in a 2-methylimidazole environment and observed distinct morphology and composition relationships. Their nickel-based derivative achieved a high specific capacitance of 698.0 F/g at 20 mV/s, attributed to its multivalent redox states and hierarchical flower-like morphology.^42^ These findings underscore the importance of understanding how secondary metal species affect the physicochemical and electrochemical properties of cobalt-based ZIF67 derivatives.

In this study, cobalt-based bimetallic compounds were synthesized in situ from ZIF67 by introducing Ni, Cu, Al, Zn, and Mn precursors in the presence of NH_4_F. Upon dissolution in water, NH_4_F releases NH_4_^+^ and F^–^ ions; the former adjusts pH and interacts with metal centers, while the latter enhances conductivity and influences crystal growth. NH_4_F is also believed to act as a steric modulator, guiding material formation toward morphologies favorable for energy storage. The resulting materials exhibit morphology and composition variations, depending on the secondary metal. A comprehensive evaluation of specific capacitance, capacity, internal resistances, rate performance, diffusion and capacitive contributions, and long-term cycling was conducted to assess their electrochemical behavior. Although numerous studies have explored ZIF67-derived materials for supercapacitors, most of them rely on post-treatment approaches. In contrast, this work applies NH_4_F as a structure-directing agent during the in situ synthesis, combined with various metal dopants, to obtain novel bimetallic ZIF67 derivatives. The electroactive materials produced herein demonstrate distinctive properties and well-characterized performance, offering insight into material design strategies for BSH applications.

## 2 Experimental Section

### 2.1 Synthesis of Cobalt-Based Bimetallic Compounds

Cobalt-based bimetallic compounds were synthesized via a simple solution process. Two precursor solutions were prepared separately and mixed under continuous stirring for 24 h. The first solution was obtained by dissolving 2.66 mmol of cobalt nitrate hexahydrate (Co­(NO_3_)_2_·6H_2_O, 99.0%, Showa) and 1.33 mmol of a secondary metal precursor in 20 mL of methanol (95%, Aecore). The second solution contained 8 mmol of 2-methylimidazole (2-melm, 99%, Acros) and 4 mmol of NH_4_F in 20 mL of methanol. After mixing, the resulting precipitate was collected by filtration, washed thoroughly with methanol, and dried at 60 °C for 24 h. The synthesized bimetallic compounds were synthesized using the secondary metal precursor of nickel nitrate hexahydrate (Ni­(NO_3_)_2_·6H_2_O, 99%, Acros), copper nitrate trihydrate (Cu­(NO_3_)_2_·3H_2_O, 99%, Acros), aluminum nitrate nonahydrate (Al­(NO_3_)_3_·9H_2_O, 99.0%, Showa), zinc nitrate hexahydrate (Zn­(NO_3_)_2_·6H_2_O, 99.0%, Showa), and manganese nitrate tetrahydrate (Mn­(NO_3_)_2_·4H_2_O, 99.0%, Showa). The synthesized bimetallic compounds were designated as CoNi, CoCu, CoAl, CoZn, and CoMn, corresponding to the use of Ni, Cu, Al, Zn, and Mn precursors, respectively. The electrode fabrication process, assembly of BSHs, and characterization techniques are detailed in the Supporting Information (SI).

### 2.2 Electrode Preparation and Hybrid Device Assembly

The working electrodes were prepared by depositing a composite slurry onto nickel foam substrates. The slurry consisted of a cobalt-based bimetallic material, carbon black, and a poly­(vinylidene fluoride) (PVdF) binder (Polysciences, Inc.) in a mass ratio of 7:2:1, with *N*-methylpyrrolidone (NMP, 100%, Echo) serving as the dispersing solvent. For the negative electrode, a commercial reduced graphene oxide (rGO) paste (Ultraphene, Nitronix) was applied onto nickel foam by a dip-coating process. The resulting battery–supercapacitor hybrid (BSH) was constructed by using the selected bimetallic compound as the positive electrode and rGO as the negative electrode, with 3 M KOH as the electrolyte.

### 2.3 Structural and Electrochemical Analysis

Surface features of the samples were analyzed by using field-emission scanning electron microscopy (FE-SEM, Nova NanoSEM 230, FEI, USA). Crystallographic structure and chemical composition were identified through X-ray diffraction (XRD, X’Pert3 Powder, PANalytical) and X-ray photoelectron spectroscopy (XPS, VG Scientific ESCALAB 250) using Al Kα radiation. Electrochemical behavior was studied in a conventional three-electrode cell, employing a platinum wire as the counter electrode and a Ag/AgCl electrode as the reference. Measurements including cyclic voltammetry (CV), galvanostatic charge/discharge (GCD), linear sweep voltammetry (LSV), and electrochemical impedance spectroscopy (EIS) were conducted by using a potentiostat/galvanostat system (PGSTAT 204, Autolab, Eco–Chemie) equipped with an FRA2 module. Impedance spectra were recorded under open-circuit conditions with frequency sweeping from 0.01 Hz to 100 kHz The specific capacitances were calculated by using eqs 1 and 2 from the cyclic voltammetry (CV) and galvanostatic charge/discharge (GC/D) curves, respectively. The energy density (*E*) and power density (*P*) were calculated by using eqs 3 and 4, respectively.^43–45^

$$C_{F}=\frac{\int IdV}{v\times \Delta V}$$ 1

$$C_{F}=\frac{I\times t}{\Delta V}$$ 2

$$E=\frac{1}{2}\frac{1}{3.6}C_{F}\times \Delta V^{2}$$ 3

$$P=\frac{E}{t}$$ 4

where *C*_F_ is specific capacitance (F/g), *v* is the scan rate (V/s), Δ*V* is the potential window (V), *E* is energy density (Wh/kg), and P is power density (W/kg).

## 3 Results and Discussion

### 3.1 Material Analysis of Bimetallic Compounds

The morphology of electroactive materials plays a critical role in determining their energy storage performance. To evaluate morphological variations induced by different metal species, SEM images of CoNi, CoCu, CoAl, CoZn, and CoMn are, respectively, presented in Figure 1a–e. Representative photographs of the powders are shown as insets to illustrate color variations among the samples. The CoNi sample exhibits a hybrid morphology composed of aggregated nanosheets and nanoparticles. Notably, some nanosheets display hexagonal geometries (highlighted in yellow), suggesting partial structural inheritance from the parent ZIF67 framework.^35^ The CoCu compound shows vertically stacked nanosheets forming layered bulk architectures with intersheet spacings of approximately 80 nm, as measured by ImageJ. In contrast, the CoAl sample reveals a network of interconnected nanoparticles with an average diameter around 100 nm. The CoZn material displays large, compact bulk structures with a rigid morphology. For CoMn, the formation of fine, loosely packed particles is observed. These morphological differences reflect the significant influence of the incorporated secondary metal on the nucleation and growth behavior during synthesis. In terms of appearance, all compounds display shades of purple, but their color depth varies. CoNi, CoCu, and CoZn appear dark purple; CoAl shows a lighter purple; and CoMn looks more like pink. These color differences likely originate from variations in the elemental composition and oxidation state. To further investigate composition, EDX spectra of CoNi, CoCu, CoAl, CoZn, and CoMn are shown in Figure S1a–e in the SI, respectively. All spectra contain signals from Co, O, C, and Si, confirming the integration of cobalt and the formation of oxygen-containing species. The Si signal arises from the underlying silicon wafer substrate. Additionally, each sample exhibits characteristic peaks for its respective secondary metal, Ni, Cu, Al, Zn, or Mn, confirming the successful synthesis of cobalt-based bimetallic compounds. These morphological differences can be attributed to the distinct ionic radii, hydrolysis rates, and coordination preferences of the incorporated secondary metals. For instance, Ni^2+^ and Cu^2+^ ions may promote anisotropic growth due to their stronger coordination with 2-methylimidazole and favorable complexation with NH_4_F, leading to sheet-like structures. In contrast, the Al^3+^ and Zn^2+^ ions tend to hydrolyze rapidly and form amorphous or densely packed particles. The Mn^2+^ with its relatively weaker interaction results in loosely packed small particles.

**Figure 1:**
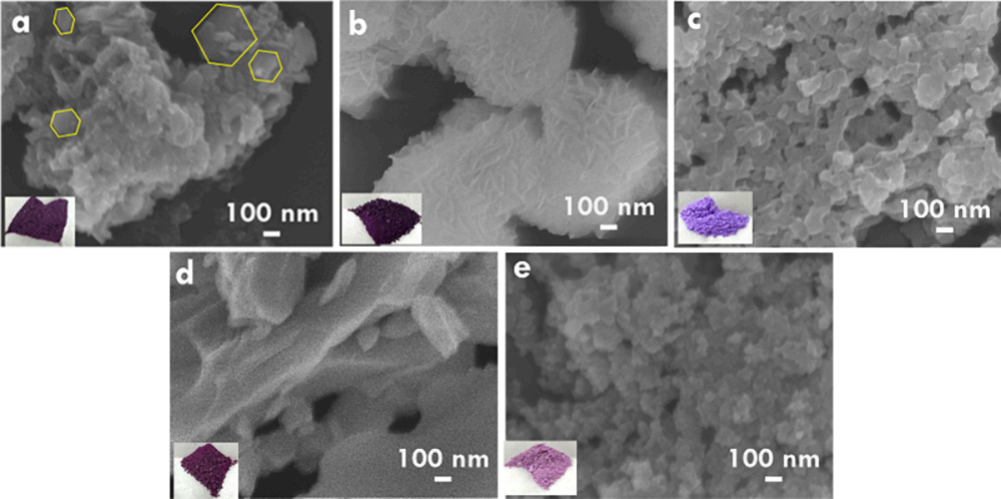
SEM images of (a) CoNi, (b) CoCu, (c) CoAl, (d) CoZn, and (e) CoMn.

The crystalline phases of the bimetallic compounds were further analyzed by XRD patterns, as shown in Figure 2a–e for CoNi, CoCu, CoAl, CoZn, and CoMn, respectively. Three standard patterns were presented in the XRD pattern of CoNi for comparison, including Ni­(OH)_2_ (JCPDS no. 074-2075),^46^ Co­(OH)F (JCPDS no. 050-0827),^47^ and Co­(OH)_2_ (JCPDS no. 074-1057).^48^ The strong reflections at approximately 19.2, 33.0, and 38.5° are assigned to Ni­(OH)_2_ and Co­(OH)_2_, while the minor peak near 35.0° corresponds to Co­(OH)­F. These results indicate that CoNi consists mainly of metal hydroxides with a partial contribution from hydroxyfluorides, likely originating from the NH_4_F additive. The XRD pattern of CoCu also includes three standard patterns of CuF_2_·2H_2_O (JCPDS no. 006-0143), CoF_2_·0.5H_2_O (JCPDS no. 040-0598), and Co­(OH)_2_ (JCPDS no. 074-1057). Prominent peaks at 16.4, 30.2, and 35.6° confirm the formation of cobalt fluoride and cobalt hydroxide, with additional weak peaks attributed to copper fluoride species. Four standard patterns were included in the XRD pattern of CoAl, including AlOOH (JCPDS no. 074-1895),^49^ AlF_3_·H_2_O (JCPDS no. 072-1117), Al_2_O_3_ (JCPDS no. 073-2294), and Co­(OH)_2_ (JCPDS no. 074-1057). The diffraction peaks are observed at 14.6, 22.3, 39.5, and 45.0°, consistent with AlOOH, AlF_3_·H_2_O, Al_2_O_3_, and Co­(OH)_2_, respectively. The predominant phase is AlOOH, while signals from cobalt hydroxide are relatively weak, indicating that the secondary metal species dominate the phase composition. The XRD pattern of CoZn includes four standard patterns, Zn­(OH)_2_ (JCPDS no. 074-0094),^50^ CoF_2_ (JCPDS no. 003-0406), Co­(OH)­F (JCPDS no. 050-0827), and Co­(OH)_2_ (JCPDS no. 074-1057). The diffraction pattern exhibits strong peaks corresponding to Zn­(OH)_2_, along with additional reflections matching those of CoF_2_, Co­(OH)­F, and Co­(OH)_2_. Peaks at 20.0, 27.5, 31.6, and 35.5° support this phase composition, suggesting a Zn-dominated structure with cobalt-based hydroxide and fluoride inclusions. Three standard patterns were included in the XRD pattern of CoMn, including MnF_2_ (JCPDS no. 024-0727),^51^ MnOOH (JCPDS no. 074-1842), and Co­(OH)_2_ (JCPDS no. 074-1057). The dominant phases are manganese fluoride and manganese hydroxide, while cobalt-based signals are present but are less intense. Overall, CoCu exhibits a cobalt-rich composition, whereas CoAl, CoZn, and CoMn are primarily composed of the secondary metal phases. These variations reflect the metal-dependent phase formation behaviors under the influence of NH_4_F and 2-methylimidazole, which likely modulate the coordination affinity of hydroxide and fluoride ions with different metal cations. To minimize the issues of low crystallinity and impurity presence observed in these composites, careful control of synthesis parameters, such as reaction temperature, time, and precursor purity, is essential. The incorporation of structure-directing agents like NH_4_F plays a crucial role in promoting the formation of more uniform and crystalline phases, thereby limiting the introduction of impurities. Additionally, thorough washing steps after synthesis help remove residual unreacted species and byproducts, contributing to improved phase purity. These combined strategies ensure enhanced crystallinity and material quality, which are critical for achieving a superior electrochemical performance.

**Figure 2:**
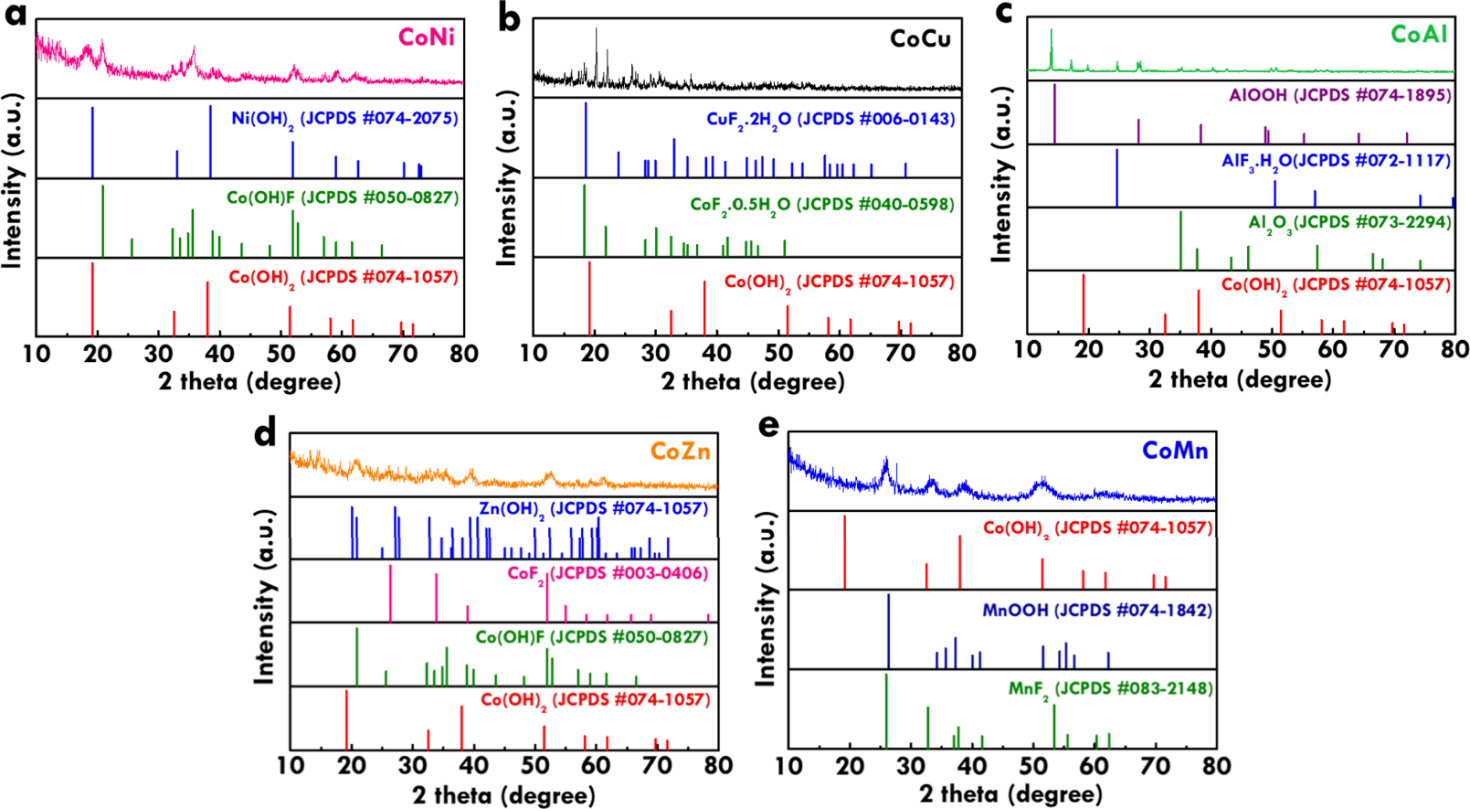
XRD patterns of (a) CoNi, (b) CoCu, (c) CoAl, (d) CoZn, and (e) CoMn.

To investigate the chemical states and surface composition of the bimetallic compounds, we conducted X-ray photoelectron spectroscopy (XPS). Figure S2a–e in the SI presents the survey spectra of CoNi, CoCu, CoAl, CoZn, and CoMn, respectively. All samples exhibit peaks corresponding to Co, O, C, N, and F, confirming the formation of cobalt-containing compounds associated with oxygen- and fluorine-based species. Nitrogen likely originates from residual NH_4_^+^, while carbon may result from ambient contamination or precursor residues. Element-specific signals for Ni, Cu, Al, Zn, and Mn further verified successful incorporation of the secondary metals. High-resolution Co 2p spectra of CoNi, CoCu, CoAl, CoZn, and CoMn are respectively provided in Figure S2f–j in the SI. Two main spin–orbit doublets are characterized at approximately 780.1 eV for Co 2p_3/2_ and 795.2 eV for Co 2p_1/2_. These peaks can be deconvoluted to Co^2+^, Co^3+^, and satellite (Sat.) peaks. Similar binding energies for all samples suggest that the local chemical environment of cobalt remains largely unaffected by the nature of the secondary metal. To further assess the chemical states of the secondary metals, their corresponding high-resolution spectra are shown in Figure 3a–e. For CoNi, the Ni 2p spectrum presents 2p_3/2_ and 2p_1/2_ peaks at around 855.8 and 873.6 eV, respectively, with satellite peaks at approximately 861.0 and 879.1 eV, indicating the coexistence of the Ni^2+^ and Ni^3+^ species. The comparable intensity and background noise of the Ni and Co signals imply that both elements are present in similar quantities, consistent with the phase composition identified by XRD. For CoCu, the 2p_3/2_ peak is observed at 932.6 eV, and is accompanied by a Cu–O peak at 934.3 eV and satellite features near 941.7 eV. The Cu 2p_1/2_ peak appears at approximately 952.5 eV. The higher background noise in the Cu 2p region compared to Co 2p suggests a relatively lower copper content, which is also supported by XRD data. For CoAl, the Al 2p spectrum exhibits a single peak at 74.3 eV, which corresponds to Al_2_O_3_. The relatively low intensity of the Co 2p signal in this sample further indicates that aluminum-based components dominate the surface. For CoZn, the Zn 2p spectrum presents two peaks at approximately 1021.8 eV (2p_3/2_) and 1044.9 eV (2p_1/2_), indicating the presence of Zn^2+^ species. Although the Zn signal is weaker than Co in XPS, XRD results suggest a more Zn-rich bulk phase, implying that cobalt may be more concentrated near the surface due to the surface-sensitive nature of XPS. For CoMn, the Mn 2p_3/2_ and 2p_1/2_ peaks are located at 641.8 and 653.5 eV, respectively. Deconvolution indicates the coexistence of Mn^3+^ and Mn^4+^ oxidation states. The stronger Mn signal compared to that of Co indicates that manganese-based compounds are dominant, consistent with the XRD observations. Additional insights are obtained from the N 1s, F 1s, and O 1s spectra. The N 1s spectra of CoNi, CoCu, CoAl, CoZn, and CoMn are respectively shown in Figure S2k–o in the SI. The peaks are corresponding to C–N–C (∼399.3 eV), C=N–C (∼400.6 eV), and N–O (∼402.3 eV), while the N–O peak is absent in the CoAl spectrum. This may reflect different nitrogen bonding environments or surface decomposition behaviors specific to the Al-based system. The F 1s spectra of CoNi, CoCu, CoAl, CoZn, and CoMn are respectively shown in Figure S3a–e in the SI. A main peak at 684.9 eV is featured, which is attributed to metal–fluorine (M-F) bonding. Notably, the lower signal intensity in CoNi and CoCu suggests reduced fluorine incorporation compared with other samples. Figure S3f–j in the SI shows the O 1s spectra of CoNi, CoCu, CoAl, CoZn, and CoMn, respectively. The peaks were centered at 529.9 eV for lattice oxygen (M-O), 531.1 eV for oxygen vacancies (O_V_), and 532.5 eV for adsorbed oxygen (O_ads_). The absence of the O_ads_ peak in CoZn and CoMn combined with a high proportion of O_V_ in CoMn indicates significant variation in surface defect density across samples. The trend in oxygen vacancy abundance follows the order CoMn > CoAl > CoNi > CoCu > CoZn. Moderate oxygen vacancy concentrations are known to enhance redox activity by providing active sites, although excessive vacancies may compromise the conductivity. Therefore, the observed variation suggests that CoMn may exhibit enhanced redox behavior, while CoZn may provide greater structural stability. Different metals, such as Mn, Al, Ni, Cu, and Zn, affect the coordination geometry, bond strengths, and defect formation energies during synthesis, which leads to varying degrees of oxygen vacancy formation. For example, Mn and Al tend to induce higher lattice distortion or strain, promoting greater oxygen vacancy concentration, while Zn tends to stabilize the lattice and suppress vacancy formation. Additionally, the presence of fluorides and hydroxides, as well as the use of NH_4_F as a structure-directing agent, further modulates the defect chemistry by altering the bonding environment and lattice parameters. These variations result in distinct oxygen vacancy abundances. The XPS analysis sufficiently demonstrates the variation in oxygen vacancies that correlates with the electrochemical behavior observed.

**Figure 3:**
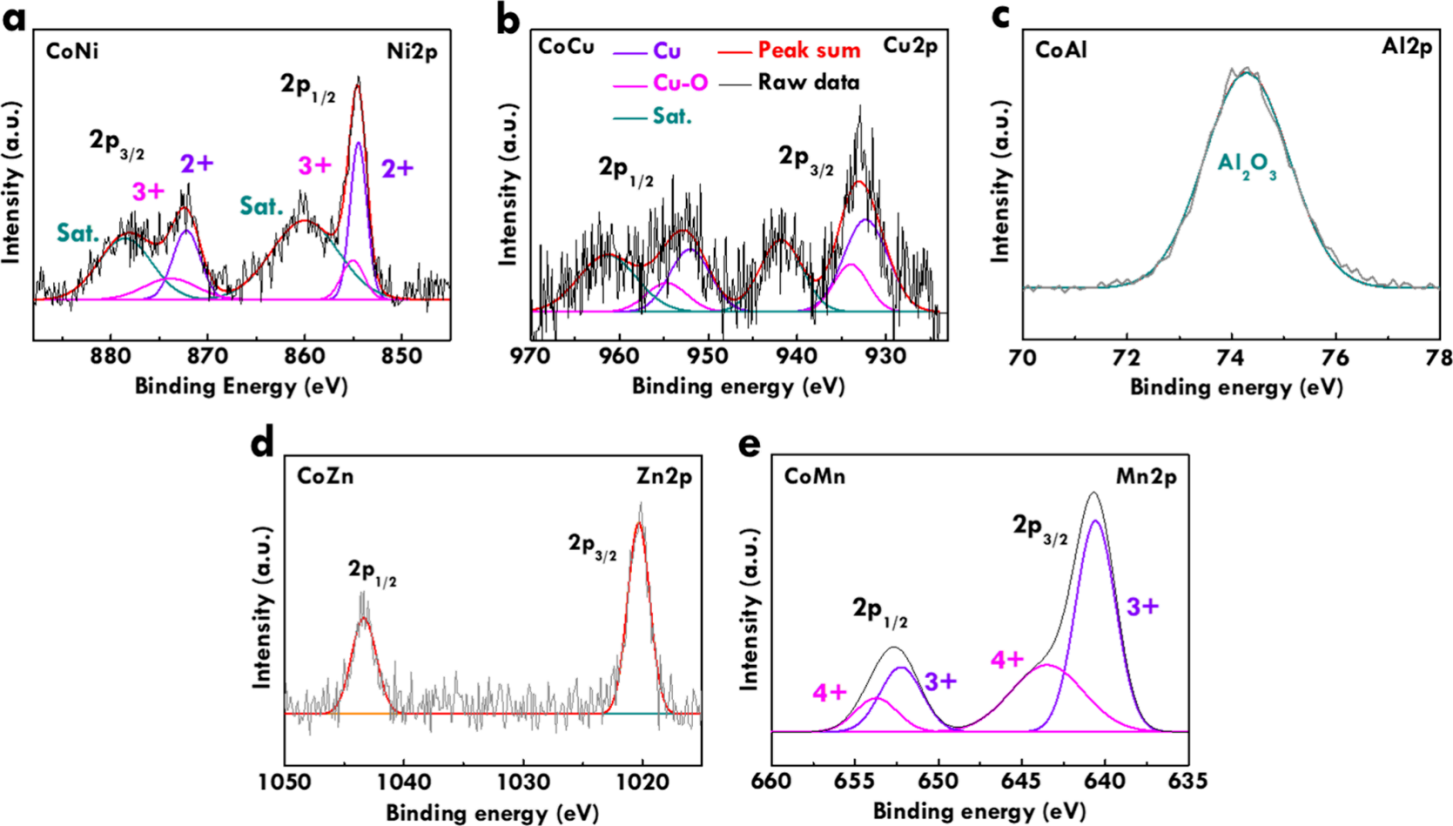
(a) Ni 2p spectra of CoNi, (b) Cu 2p spectra of CoCu, (c) Al 2p spectra of CoAl, (d) Zn 2p spectra of CoZn, and (e) Mn 2p spectra of CoMn.

### 3.2 Electrochemical Performance of Bimetallic Compounds

The electrochemical performance of the synthesized cobalt-based bimetallic compounds was evaluated by cyclic voltammetry (CV), galvanostatic charge–discharge (GC/D), and electrochemical impedance spectroscopy (EIS). Figure 4a presents the CV curves measured at 20 mV/s. Owing to differences in composition and redox-active species, each electrode exhibits unique redox behavior and peak configurations. All compounds contain Co­(OH)_2_ as a common component, which participates in the redox reaction Co­(OH)_2_ + OH^–^ → CoOOH + H_2_O. The CoNi electrode shows an oxidation peak at 0.20 V vs Ag/AgCl and two reduction peaks at 0.06 and 0.38 V vs Ag/AgCl. These peaks are attributed to the combined contributions of Co­(OH)_2_ and Ni­(OH)_2_, as confirmed by XRD, which revealed comparable quantities of both cobalt- and nickel-based phases. The superior capacitance of CoNi arises from the synergistic redox activity between Co and Ni species, where Ni­(OH)_2_ enhances the electronic conductivity and provides additional reversible redox sites, thus boosting the charge storage and kinetics. For the CoCu electrode, three oxidation peaks appear at 0.11, 0.29, and 0.37 V vs Ag/AgCl, accompanied by three reduction peaks at −0.02, 0.18, and 0.35 V vs Ag/AgCl. These redox pairs are likely associated with CuF_2_·2H_2_O, CoF_2_·0.5H_2_O, and Co­(OH)_2_. CoCu exhibits relatively high conductivity due to copper species, which facilitates fast electron transport. The vertically stacked nanosheet morphology increases the surface area and electrolyte access, supporting effective redox reactions mainly dominated by Co­(OH)_2_. This combination leads to good capacitance and rate performance, albeit slightly lower than CoNi due to less pronounced redox synergy. The CoAl electrode exhibits two oxidation peaks at 0.37 and 0.42 V vs Ag/AgCl and two reduction peaks at 0.29 and 0.23 V vs Ag/AgCl, attributed to Co­(OH)_2_ and Al-containing phases. The limited capacitance of CoAl stems from the relatively inert nature of aluminum hydroxide and fluoride species, which contribute fewer redox-active sites. Moreover, the interconnected nanoparticle morphology results in higher internal resistance, restricting ion diffusion and electronic transport, thereby reducing electrochemical performance. The CoZn electrode shows three pairs of redox peaks, with oxidation features at 0.08, 0.34, and 0.45 V vs Ag/AgCl and reduction features at −0.02, 0.20, and 0.37 V vs Ag/AgCl. These peaks are associated with Zn­(OH)_2_, CoF_2_, Co­(OH)­F, and Co­(OH)_2_. Notably, some large peaks are interpreted as convolution of closely spaced redox signals. The large, compact bulk morphology of CoZn contributes to enhanced structural stability during cycling, which supports better performance at high scan rates and power densities. Although zinc species offer moderate redox activity, the solid structure reduces oxygen vacancies, favoring stability over capacity. This morphology and composition combination explain the moderate capacitance but excellent rate capability and cycling durability of CoZn. The CoMn electrode reveals two redox couples with oxidation at 0.31 and 0.36 V vs Ag/AgCl and reduction at 0.06 and 0.16 V vs Ag/AgCl, corresponding to Co­(OH)_2_, MnOOH, and MnF_2_. CoMn features high oxygen vacancy concentrations and loosely packed fine particles, which increase active sites and facilitate electrolyte penetration. The manganese species contribute additional redox reactions, enhancing the pseudocapacitive behavior. However, the less compact structure may slightly compromise the conductivity, balancing moderate capacitance with improved redox kinetics. Table 1 summarized the *C*_F_ values of CoNi, CoCu, CoAl, CoZn, and CoMn electrodes calculated from the CV curves.^52^ The *C*_F_ values of 997.3, 967.3, 382.3, 816.3, and 785.0 F/g were respectively obtained for the CoNi, CoCu, CoAl, CoZn, and CoMn electrodes, corresponding to the specific capacities of 748.0, 725.5, 286.7, 612.2, and 588.8 C/g. To compare, a previous study without NH_4_F yielded a CoNi-derived ZIF67 with a *C*_F_ of only 490.4 F/g.^35^ The superior capacitance of CoNi is attributed to the synergistic redox activity of Ni­(OH)_2_ and Co­(OH)_2_ with incorporation of NH_4_F. The slightly lower value for CoCu may result from the dominance of Co­(OH)_2_ and the favorable conductivity of the copper-based species. The nanoscale morphology of CoCu also contributes to enhanced surface accessibility. In contrast, the low capacitance of CoAl reflects the limited redox activity of aluminum hydroxide and fluoride species. CoZn and CoMn exhibit moderate *C*_F_ values, with contributions from CoF_2_ and Zn­(OH)_2_ or MnOOH, respectively. In comparison, the inclusion of NH_4_F in this work promoted the formation of Ni­(OH)_2_, Co­(OH)_2_, and Co­(OH)­F, leading to a significantly enhanced *C*_F_ of 997.3 F/g. This highlights the dual role of NH_4_F as both a pH regulator and a structure-directing agent that favors hydroxide and fluoride phase formation. Moreover, it is important to emphasize that the observed electrochemical performance cannot be solely attributed to the metal centers present in the samples. As revealed by SEM and XRD analyses, the morphology and phase composition vary significantly depending on the secondary metal species incorporated during the synthesis. For instance, the CoNi sample features a hybrid morphology of nanosheets and nanoparticles, which can facilitate ion diffusion and provide abundant active sites, thus enhancing charge storage capacity. In contrast, the compact bulk structures of CoZn or the loosely packed particles of CoMn influence ion transport kinetics differently and result in varied capacitance and rate capabilities. Similarly, the dominance of certain crystalline phases such as hydroxides, fluorides, or oxides, as indicated by XRD, affects the redox activity and electrical conductivity of the electrodes. The presence of multiple metal hydroxide and fluoride phases, modulated by NH_4_F as a structure-directing agent, contributes to the complexity of redox processes and the overall electrode behavior. Therefore, both the morphology and precise compositional makeup, including phase distribution and crystallinity, synergistically determine the electrochemical responses observed. These factors should be considered alongside metal center contributions to fully understand and optimize the energy storage performance of these bimetallic compounds. The GC/D curves of CoNi, CoCu, CoAl, CoZn, and CoMn electrodes measured at 4 A/g are further shown in Figure 4b, with redox plateaus that closely align with the redox peaks observed in their CV curves. The *C*_F_ values of the cobalt-based bimetallic compounds are calculated based on the previous study,^53^ as listed in Table 1. The *C*_F_ values of 568.3, 543.1, 191.6, 464.7, and 417.6 F/g corresponding to the specific capacities of 369.4, 353.0, 124.5, 302.1, and 271.4 C/g are obtained for CoNi, CoCu, CoAl, CoZn, and CoMn electrodes, respectively. These results again confirm the superior charge storage capability of CoNi. The GC/D curves also allow for a comparison of internal resistance behavior via analysis of the IR drop, which appears as a sudden voltage drop at the beginning of each discharge curve. The IR drop is a reflection of the equivalent series resistance (ESR), including contributions from the electrolyte, electrode material, and the interface between the electrode and current collector. Among the five samples, CoNi and CoCu electrodes exhibit relatively small IR drops, indicating low internal resistance and efficient charge transport. In contrast, CoAl displays a more pronounced IR drop, suggesting a higher internal resistance and poorer conductivity, which can hinder rapid ion transport and reduce the overall energy efficiency of the device. CoZn and CoMn show moderate IR drops, aligning with their intermediate *R*_S_ values and redox behavior. In addition to the voltage drop, the shape of the GC/D profiles reveals the reversibility of the redox reactions. CoNi and CoCu electrodes demonstrate well-defined and symmetric charge/discharge curves with distinguishable plateaus, which are indicative of pseudocapacitive behavior driven by Faradaic redox processes. The sloping discharge regions observed for CoAl and CoMn suggest a less pronounced redox contribution and possibly a higher contribution from the electric double-layer capacitance or kinetically slower redox processes. The plateau lengths also correlate with the charge storage capacity with CoNi exhibiting the longest discharge duration, supporting its superior specific capacitance. Overall, the analysis of GC/D curves, including IR drop, symmetry, and plateau clarity, reinforces the conclusion that CoNi provides the most favorable charge storage characteristics among the synthesized bimetallic compounds, while the lower performance of CoAl is primarily constrained by its inferior conductivity and less active redox species.

**Figure 4:**
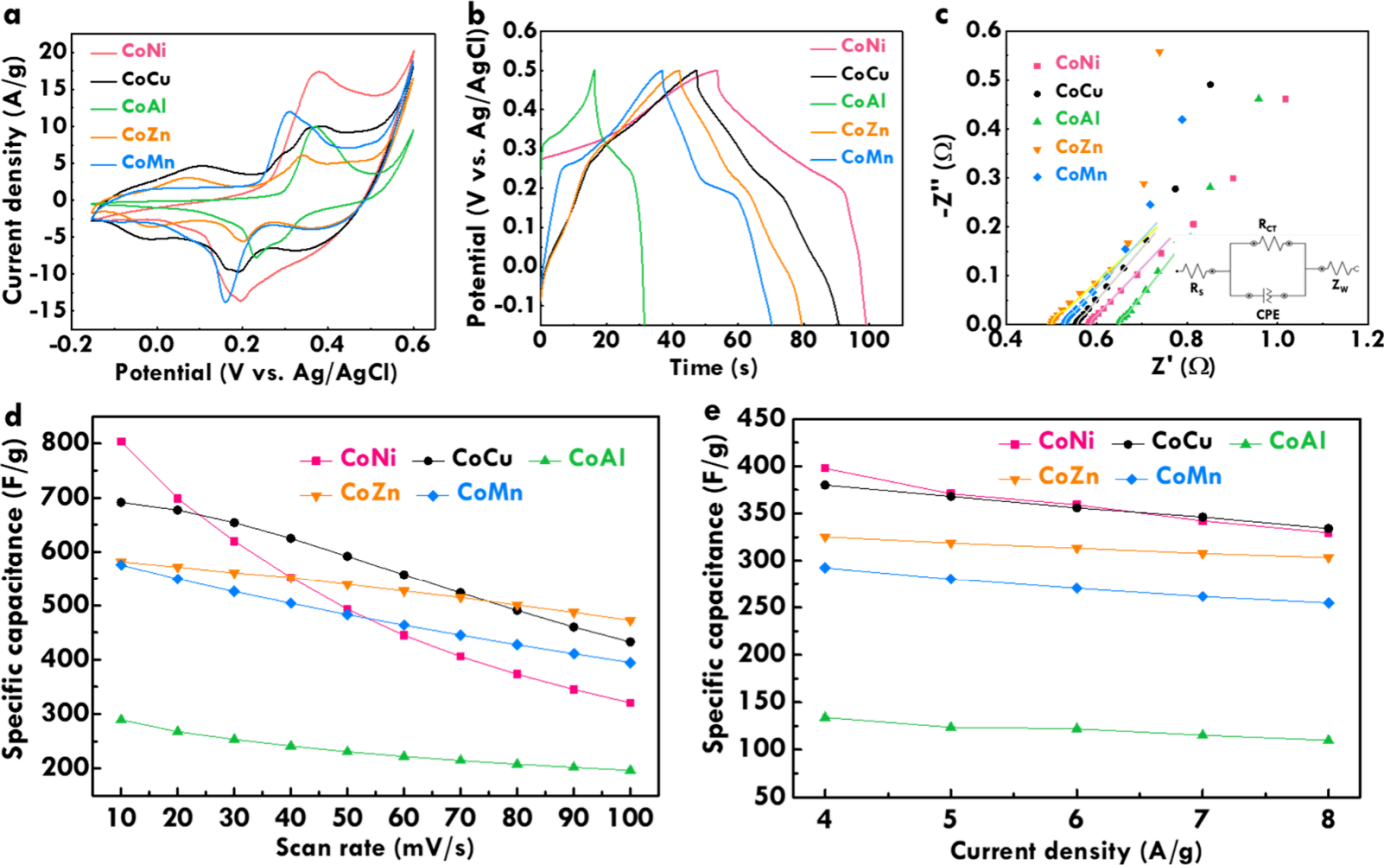
(a) CV curves at 20 mV/s, (b) GC/D curves at 4 A/g, and (c) Nyquist plot and equivalent circuit; relationships of specific capacitance to (d) the scan rate and (e) the current density for the CoNi, CoCu, CoAl, CoZn, and CoMn electrodes.

**Table 1:** *C*_F_ Value, Capacity, *R*_S_, and *R*_ct_ Values of CoNi, CoCu, CoAl, CoZn, and CoMn Electrodes

**electrode**	***C***_**F**_ **(F/g)** ^a^	**capacity** **(C/g)** ^a^	***C***_**F**_ **(F/g)** ^b^	**capacity** **(C/g)** ^b^	***R***_**S**_ **(Ω)**	***R***_**ct**_ **(Ω)**
CoNi	997.3	748.0	568.3	369.4	0.58	0.06
CoCu	967.3	725.5	543.1	353.0	0.55	0.10
CoAl	382.3	286.7	191.6	124.5	0.65	0.20
CoZn	816.3	612.2	464.7	302.1	0.50	0.12
CoMn	785.0	588.8	417.6	271.4	0.53	0.14

a These values were calculated using the CV curves at 20 mV/s.

b These values were calculated using the GC/D curves at 4 A/g.

Electrochemical impedance spectroscopy was used to assess the charge transport properties. The resistances such as the solution resistance (*R*_S_) and charge-transfer resistance (*R*_ct_) of the cobalt-based bimetallic compound electrodes are analyzed by the Nyquist plot, as presented in Figure 4c. The equivalent circuit is shown in this figure for fitting the resistances.^54^ The *R*_S_ and *R*_ct_ values of CoNi, CoCu, CoAl, CoZn, and CoMn electrodes are listed in Table 1 for comparison. The *R*_S_ value is inversely proportional to the conductivity of the electrodes. The *R*_S_ values of 0.58, 0.55, 0.65, 0.50, and 0.53 Ω are respectively obtained for the CoNi, CoCu, CoAl, CoZn, and CoMn electrodes. Similar *R*_S_ values are obtained for all bimetallic compounds due to the consistent use of highly conductive Ni foam as the substrate. The *R*_ct_ value is an indicator for the charge-transfer resistance between the electrode and electrolyte. The *R*_ct_ values of 0.06, 0.10, 0.20, 0.12, and 0.14 Ω are respectively obtained for the CoNi, CoCu, CoAl, CoZn, and CoMn electrodes. The CoNi electrode shows a much smaller *R*_ct_ value than those of other cobalt-based bimetallic compounds, indicating enhanced redox kinetics due to the presence of multiple oxidation states of Co and Ni.

To further assess rate capability, CV and GC/D measurements were performed under various scan rates and current densities. The CV curves of CoNi, CoCu, CoAl, CoZn, and CoMn electrodes were measured using different scan rates, as respectively shown in Figure S4a–e in the SI. All CV curves retain symmetric shapes and small peak separations even at higher scan rates, indicating good reversibility. The GC/D curves of CoNi, CoCu, CoAl, CoZn, and CoMn electrodes were also measured by using different current densities, as respectively shown in Figure S4f–j in the SI. Similarly, GC/D curves remain nearly triangular and symmetrical, confirming efficient charge–discharge behavior under rapid operation. To quantitatively evaluate the high-rate performance, the relationships between the *C*_F_ values and the conditions for measuring CV curves for CoNi, CoCu, CoAl, CoZn, and CoMn electrodes are presented in Figure 4d. As expected, capacitance declines with an increasing scan rate due to kinetic limitations. CoNi exhibits the highest capacitance at low scan rates (10 and 20 mV/s), but its performance deteriorates more rapidly under high-rate conditions. At 100 mV/s, CoZn displays the highest *C*_F_, suggesting improved tolerance to fast charge and discharge. On the other hand, the relationships between the C_F_ values and the conditions for measuring GC/D curves for CoNi, CoCu, CoAl, CoZn, and CoMn electrodes are presented in Figure 4e. More gradual declines are found compared with the CV results. CoNi and CoCu retain high capacitances up to 8 A/g, whereas CoZn shows better performance under extreme conditions. The solid, compact morphology of CoZn likely facilitates structural stability and ion accessibility under stress, making it a promising material for high-power applications.

To further elucidate the charge storage mechanism, we examined the contributions from diffusion-controlled and capacitive processes. The CV curves measured at 10 mV/s for CoNi, CoCu, CoAl, CoZn, and CoMn electrodes are respectively shown in Figure 5a–e. Based on these data, the relative proportions of capacitive and diffusion-controlled contributions across various scan rates were quantitatively evaluated, as illustrated in Figure 5f–j for CoNi, CoCu, CoAl, CoZn, and CoMn. The CoNi and CoCu electrodes exhibit relatively low capacitive contributions at all scan rates, indicating that a larger portion of their current response arises from diffusion-limited redox reactions. This result supports their superior specific capacitance values, as higher diffusion contributions generally enhance Faradaic charge storage through redox activity. In contrast, the CoZn electrode demonstrates the highest capacitive contribution among all bimetallic electrodes, particularly at higher scan rates. This behavior aligns with its excellent capacitance retention under high-rate conditions. The dominance of capacitive charge storage in CoZn suggests a stronger electric double-layer capacitance (EDLC) component, which favors fast surface-controlled charge accumulation and contributes to its structural stability and reversibility during rapid cycling. The CoMn and CoAl electrodes show intermediate behavior, with balanced contributions from both charge storage mechanisms.

**Figure 5:**
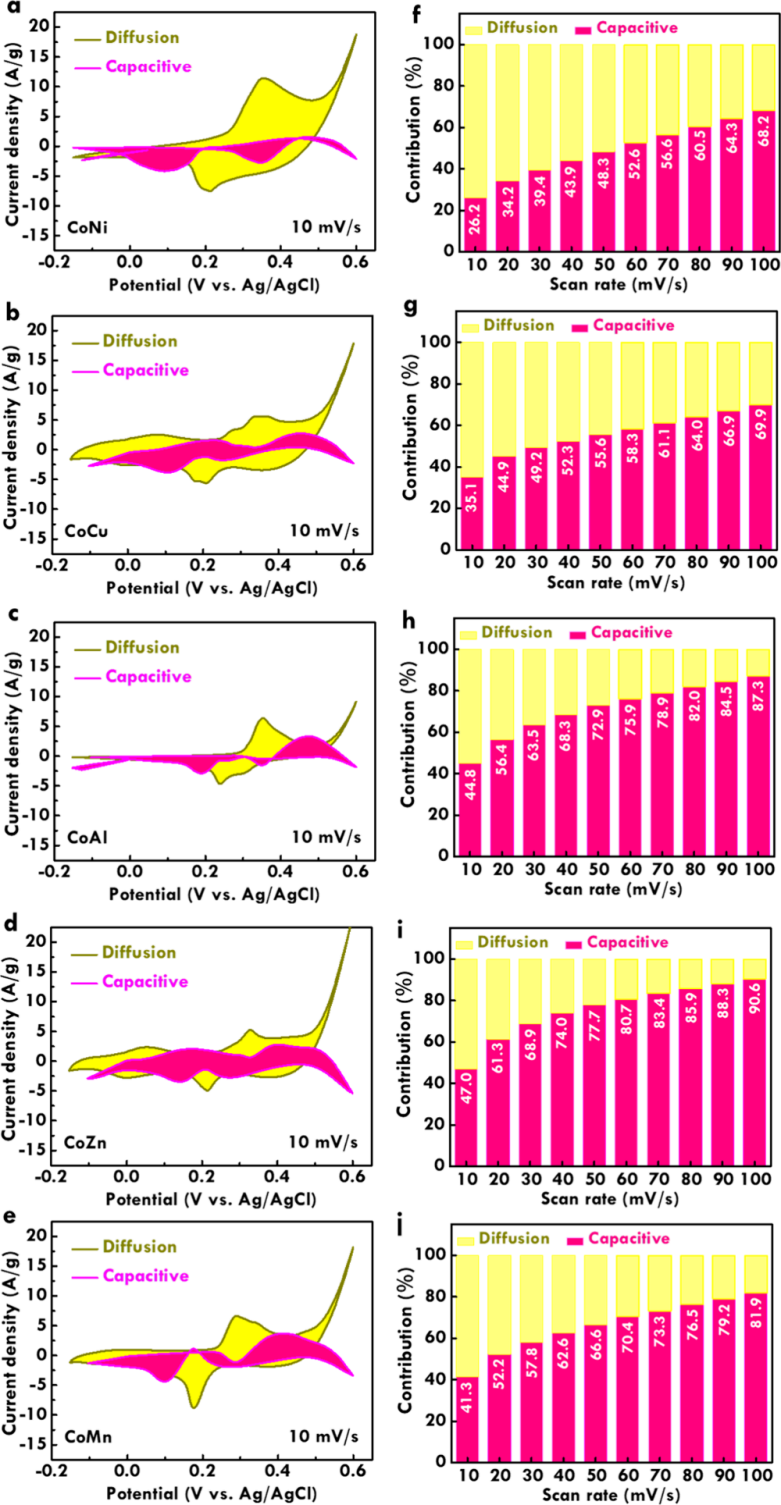
Diffusion/capacitive CV curves of (a) CoNi, (b) CoCu, (c) CoAl, (d) CoZn, and (e) CoMn; diffusion/capacitive contributions to the scan rate of (f) CoNi, (g) CoCu, (h) CoAl, (i, top) CoZn, and (i, bottom) CoMn.

To gain further insight into electrochemical kinetics, the relationships of log­[peak current] to log­[scan rate] that were analyzed based on the power law equation *i* = *av* ^*b*^, where *i* represents the peak current, *v* represents the scan rate, and *a* and *b* are constants,^55^ are shown in Figure 6a–e for the CoNi, CoCu, CoAl, CoZn, and CoMn electrodes, respectively. These plots are derived from the CV curves at various scan rates (Figure S4a–e in the SI). The reduction and oxidation peaks were used to evaluate the reaction kinetics using the equation with the slope of the fitting line representing the *b-*values. For the reduction peaks, the calculated *b*-values are 0.71 for CoNi, 0.80 for CoCu, 0.68 for CoAl, 0.78 for CoZn, and 0.76 for CoMn. The corresponding *b*-values for oxidation peaks are 0.53, 0.79, 0.73, 0.77, and 0.61, respectively. These *b*-values fall between 0.5 and 1.0, confirming that charge storage in all electrodes arises from a combination of capacitive and diffusion-controlled processes. Notably, the CoNi electrode exhibits the smallest *b*-values for both oxidation and reduction peaks, implying that its electrochemical behavior is more strongly governed by diffusion processes. This finding suggests that the CoNi electrode facilitates more effective ion diffusion within its porous architecture, enabling efficient electrolyte migration and redox reactions. The results complement the previous analysis and further highlight the potential of CoNi as a high-performance electrode material for energy storage applications.

**Figure 6:**
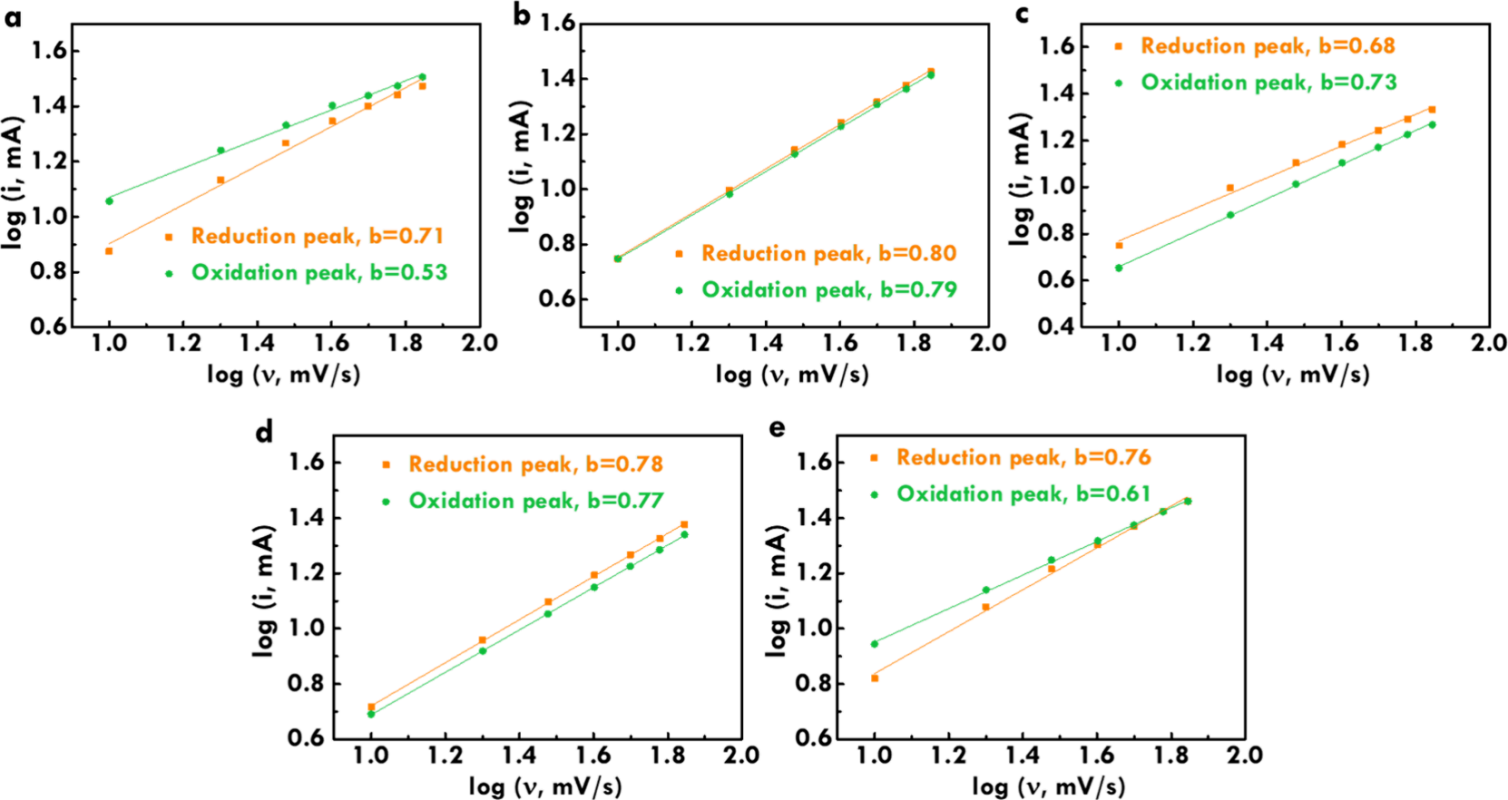
Relationships of log­[*i*] to log­[scan rate] of (a) CoNi, (b) CoCu, (c) CoAl, (d) CoZn, and (e) CoMn electrodes.

### 3.3 Electrochemical Analysis of a Battery–Supercapacitor Hybrid with a CoNi Positive Electrode and a rGO Negative Electrode

To evaluate practical applicability, a BSH was assembled using CoNi/NF as the positive electrode and rGO/NF as the negative electrode. Figure S5 in the SI shows the CV curve of the rGO electrode as the reference. Figure 7a displays CV curves measured at 20 mV/s over the potential windows from 1.1 to 1.4 V. A minor anodic current rise attributed to water oxidation is observed when the voltage window reached 1.4 V, indicating the onset of electrolyte decomposition. Therefore, 1.3 V was selected as the optimal operating potential to ensure both electrochemical performance and stability. In Figure 7b, the CV curves were recorded at different scan rates with the potential window of 1.3 V. All curves maintain similar profiles with minor peak separations even at the highest scan rate of 100 mV/s, suggesting favorable charge-transfer kinetics and high reversibility. Figure 7c shows the GC/D curves at 1.0 A/g with different potential windows. Notably, a significant increase in IR drop is observed when the voltage window reaches 1.4 V, reflecting a higher internal resistance likely due to side reactions such as water decomposition. This further supports the selection of 1.3 V as the optimal window. The IR drop, characterized by a sharp voltage decline at the beginning of the discharge curve, indicates the internal series resistance of the device. Its minimization is critical for preserving the energy efficiency and power output. At 1.3 V, the relatively small IR drop suggests a low ESR, confirming efficient charge transport across the device components. Based on this potential window, Figure 7d illustrates the GC/D curves measured at varying current densities from 0.5 to 2.5 A/g. All discharge curves exhibit high symmetry with negligible deformation, demonstrating excellent high-rate performance and minimal polarization effects under increasing current load. The relationship between the energy and power densities is summarized in the Ragone plot (Figure 7e). A maximum energy density of 9.2 Wh/kg is achieved at a power density of 375 W/kg. Even at a maximum power density of 1625 W/kg, the energy density remains 6.2 Wh/kg, indicating a well-balanced performance between energy output and rapid response which is essential for real-world energy storage systems. Cycling stability was further examined over 10,000 consecutive charge/discharge cycles at 1.0 A/g, as shown in Figure 7f. The specific capacitance increased during the initial 3000 cycles, exceeding 100% retention. This phenomenon is attributed to an activation process that is commonly observed in porous electrode systems. During the early cycling phase, repeated electrolyte penetration can lead to pore expansion, thereby increasing the accessible surface area and facilitating greater redox activity. As a result, more charges can be stored with an improved electrolyte/electrode contact. After conducting 10,000 charging and discharging cycles, the device shows a *C*_F_ retention of 83.1% and a Coulombic efficiency of 94.2%, underscoring the excellent long-term electrochemical stability of the BSH system constructed with a CoNi-based positive electrode and a rGO negative electrode. This performance highlights the promising potential of assembled BSH for practical applications. Nevertheless, it is important to recognize the intrinsic trade-off between achieving high capacitance and ensuring long-term cycling stability. While materials with high redox activity and large surface area can significantly enhance energy storage capabilities, they are also more prone to structural degradation, volume expansion, or dissolution of active species during prolonged cycling. Conversely, materials with robust structural frameworks may exhibit slightly lower capacitance but offer superior long-term durability. Therefore, in real-world energy storage systems, optimizing this balance is essential. The integration of CoNi-based compounds and rGO in the current system demonstrates a well-coordinated strategy to address this challenge by achieving both high energy performance and stable operation over extensive cycling.

**Figure 7:**
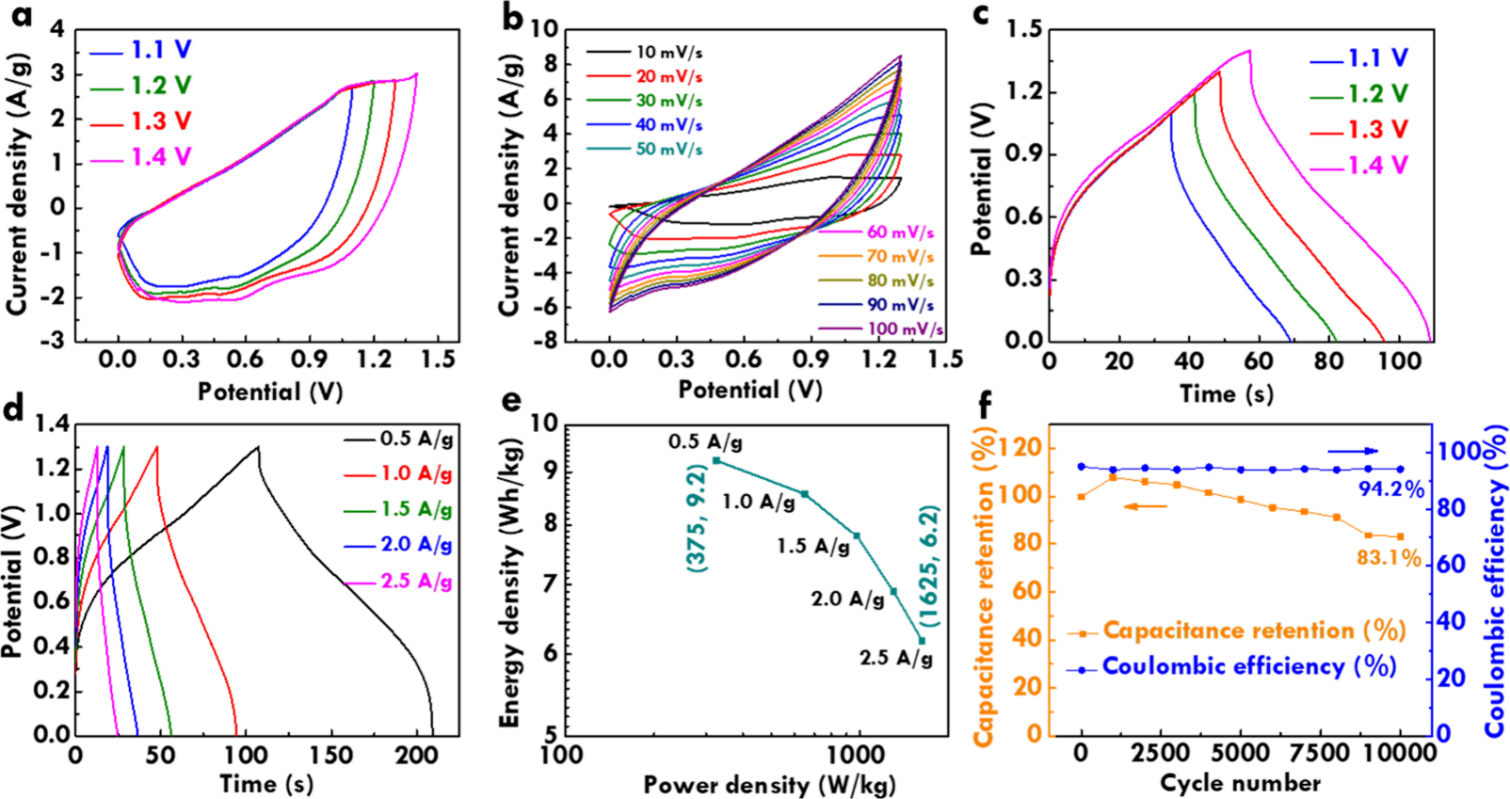
CV curves (a) measured using 20 mV/s at different potential windows and (b) measured using different scan rates at the potential window of 1.3 V; GC/D curves (c) measured using 1.0 A/g at different potential windows and (d) measured using different current densities at the potential window of 1.3 V; (e) Ragone plot and (f) plot of *C*_F_ retention and Coulombic efficiency relating to the cycle number measured at 1.0 A/g for the BSH with CoNi and rGO.

A comparison of electrochemical parameters for ammonium-directed materials reported in previous studies and this work is summarized in Table 2. The CoNi compound synthesized in this work exhibits a specific capacitance of 997.3 F/g at 20 mV/s, surpassing those reported for NH_4_F-induced ZIF67 derivatives (475.8 F/g) and ammonium phosphate-based systems such as NH_4_(Ni, Co)­PO_4_ (811.1 F/g at 1.25 A/g). In terms of device performance, the BSH of CoNi//rGO fabricated in this study achieved a competitive energy density of 9.2 Wh/kg at a power density of 375 W/kg, which exceeds 2.8 Wh/kg at 400 W/kg reported for the NH_4_F-induced ZIF67-derived system and 6.44 Wh/kg at 0.7 kW/kg for CoMoFN-based BSH. Although NH_4_(Ni, Co)­PO_4_ demonstrates a higher energy density of 20.28 Wh/kg, it was evaluated at a lower power density (189.17 W/kg), suggesting that the CoNi-based device achieves a better energy/power trade-off in high-rate conditions. The CoNi-based BSH also shows excellent long-term cycling stability with a capacitance retention of 83.1% after 10,000 cycles. This performance is comparable to other reported ammonium-directed systems, such as (Ni, Co)_2_(CO_3_)­(OH)_2_ (96.6%@10,000) and NH_4_(Ni, Co)­PO_4_ (89.1%@2000), and is better than CoMoFN (73%@6500).

**Table 2:** Partial Lists of Electrochemical Parameters for Electrodes and BSHs with Active Materials Synthesized by Ammonium-Based SDA in Previous Studies and in This Work^a^

**active material**	***C***_**F**_ **for a single electrode**	**energy density @power density**	***C***_**F**_ **retention @cycle number**	**ref.**
NH_4_(Ni, Co)PO_4_	811.1 F/g @1.25 A/g	20.28 Wh/kg @189.17 W/kg	89.1% @2000	^56^
NH_4_F-induced ZIF67 derivative	475.8 F/g @20 mV/s	2.8 Wh/kg @400 W/kg	117% @4000	^57^
(Ni, Co)_2_(CO_3_)(OH)_2_	1247 F/g @1 A/g	NA	96.6% @10,000	^58^
MB21-CoNi	763.2 F/g @20 mV/s	17.2 Wh/kg @650 W/kg	94.9% @10,000	^59^
ZIF-L18	1347.0 F/g @20 mV/s	11.96 Wh/kg @650 W/kg	83.6% @10,000	^60^
MHB5	1410.3 F/g @20 mV/s	32.40 Wh/kg @375 W/kg	77.4% @10,000	^61^
MB-450	580.2 F/g @20 mV/s	7.15 Wh/kg @250 W/kg	91% @10,000	^62^
ZIF-NBF	1527.0 F/g @20 mV/s	15.1 Wh/kg @857 W/kg	90% @5000	^43^
CoMoFN	33.7 mAh/g @20 mV/s	6.44 Wh/kg @700 W/kg	73% @6500	^63^
CoNi	997.3 F/g @20 mV/s	9.2 Wh/kg @375 W/kg	83.1% @10,000	this work

a CoMoFN = ammonium-decorated cobalt molybdenum fluoride.

## 4 Conclusions

Cobalt-based bimetallic compounds were successfully derived from ZIF67 through a simple solution method involving NH_4_F and 2-methylimidazole. The electrochemical behavior of the CoNi, CoCu, CoAl, CoZn, and CoMn electrodes was systematically investigated for application in BSHs. Morphology and composition of these Co-based compounds are strongly influenced by the incorporated secondary metal, leading to diverse structural and electrochemical features. The CoNi electrode shows the highest *C*_F_ value of 997.3 F/g at 20 mV/s, attributed to the synergistic redox activity between cobalt and nickel hydroxides and fluorides. This synergistic interaction developed under the influence of NH_4_F and 2-methylimidazole represents a novel approach not previously explored in bimetallic material synthesis. A BSH assembled using CoNi and rGO electrodes achieved a maximum energy density of 9.2 Wh/kg and a power density of 375 W/kg. Remarkable long-term cycling stability was demonstrated with a capacitance retention of 83.1% and a Coulombic efficiency of 94.2% after 10,000 cycles. Future work may focus on exploring alternative structure-directing agents or further optimizing electrode architectures to enhance the overall electrochemical performance.

## Supplementary Material



## Notes

EDX, XPS, CV, and GC/D analysis of cobalt-based bimetallic compounds (PDF)
