# Supplementary material for: Engineering Cobalt-Based Bimetallic Compounds via NH4F‑Directed ZIF67 Transformation for Battery–Supercapacitor Hybrids with Enhanced Energy Storage Performance
Source: ACS Omega. 2025 Aug 22;10(35):40089–100. doi: 10.1021/acsomega.5c04906 (PMC12423812; doi:10.1021/acsomega.5c04906)
Supplement: Supplementary file 1 [file ao5c04906_si_001.pdf]

Supporting Information (SI)

Engineering Cobalt-Based Bimetallic Compounds via NH_4F-Directed ZIF67 Transformation for Battery–Supercapacitor Hybrids with Enhanced Energy Storage Performance

Tsai-Mu Cheng[a,b,c*], Yi-An Lu[d], Pin-Chun Lee[d], Chutima Kongvarhodom[e], Sadang Husain[f], Sibidou Yougbaré[g], Hung-Ming Chen[h], and Lu-Yin Lin[d*]

[a]Graduate Institute for Translational Medicine, College of Medical Science and Technology, Taipei Medical University, Taipei 11031, Taiwan

[b]Taipei Heart Institute, Taipei Medical University, Taipei 11031, Taiwan

[c]Cardiovascular Research Center, Taipei Medical University Hospital, Taipei 11031, Taiwan

[d]Department of Chemical Engineering and Biotechnology, National Taipei University of Technology, Taipei, Taiwan

[e]Department of Chemical Engineering, King Mongkut's University of Technology Thonburi, 126 Pracha-u-thit, Toong-kru, Bangkok 10140, Thailand

[f]Department of Physics, Faculty of Mathematics and Natural Science, Lambung Mangkurat University, Banjarmasin 70124, Indonesia

[g]Institut de Recherche en Sciences de la Santé (IRSS-DRCO)/Nanoro, 03 B.P 7192, Ouagadougou 03, Burkina Faso

[h]Gingen technology Co., LTD., Rm. 7, 10F., No.189, Sec. 2, Keelung Rd., Xinyi Dist., Taipei 11054, Taiwan

[*]Corresponding author (T.M. Cheng): E-mail: tmchent@tmu.edu.tw

[*]Corresponding author (L.Y. Lin): E-mail: lylin@ntut.edu.tw

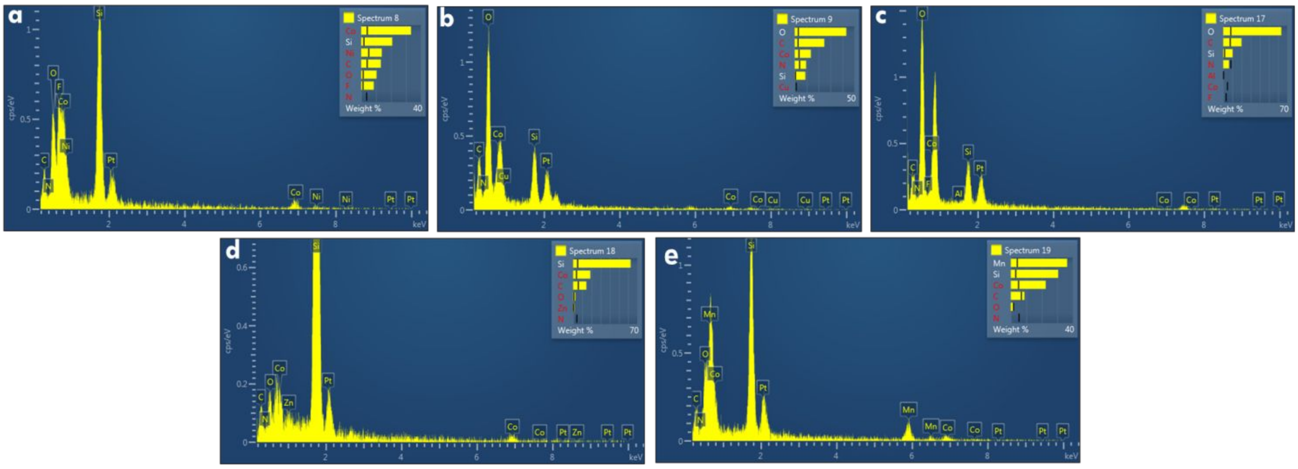

Figure S1 EDX spectra of (a) CoNi, (b) CoCu, (c) CoAl, (d) CoZn and (e) CoMn.

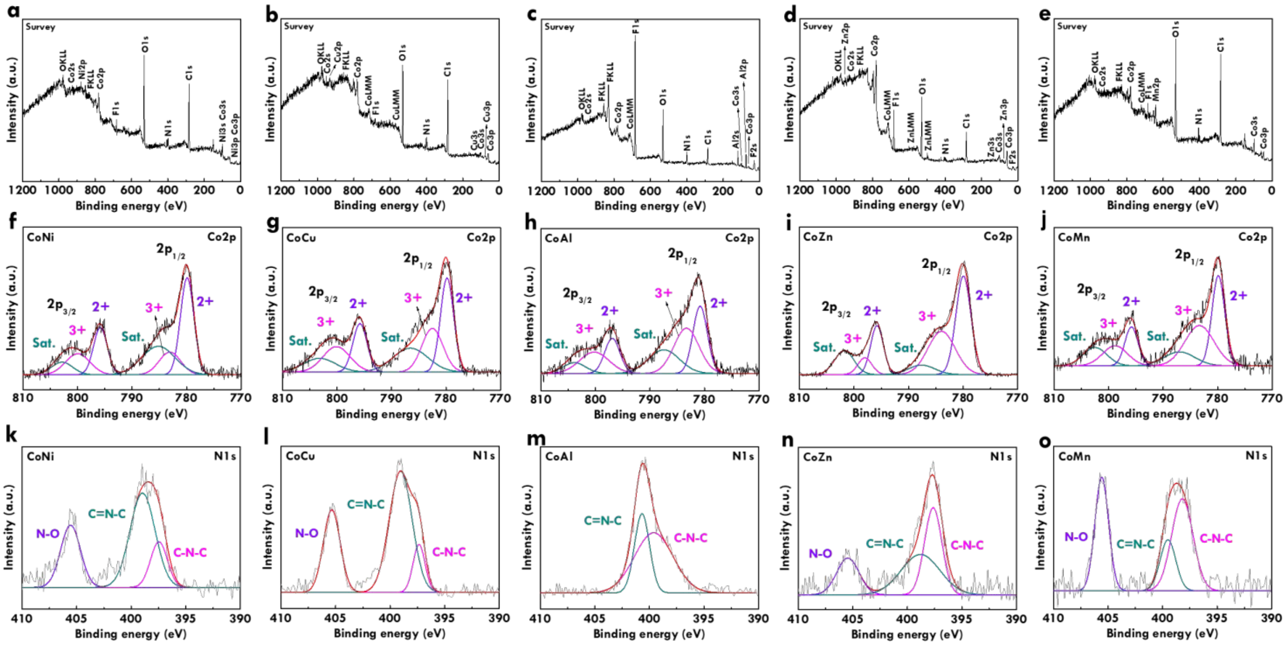

Figure S2 The XPS survey spectra of (a) CoNi, (b) CoCu, (c) CoAl, (d) CoZn and (e) CoMn; the Co2p spectra of (f) CoNi, (g) CoCu, (h) CoAl, (i) CoZn and (j) CoMn; N1s spectra of (k) CoNi, (l) CoCu, (m) CoAl, (n) CoZn and (o) CoMn.

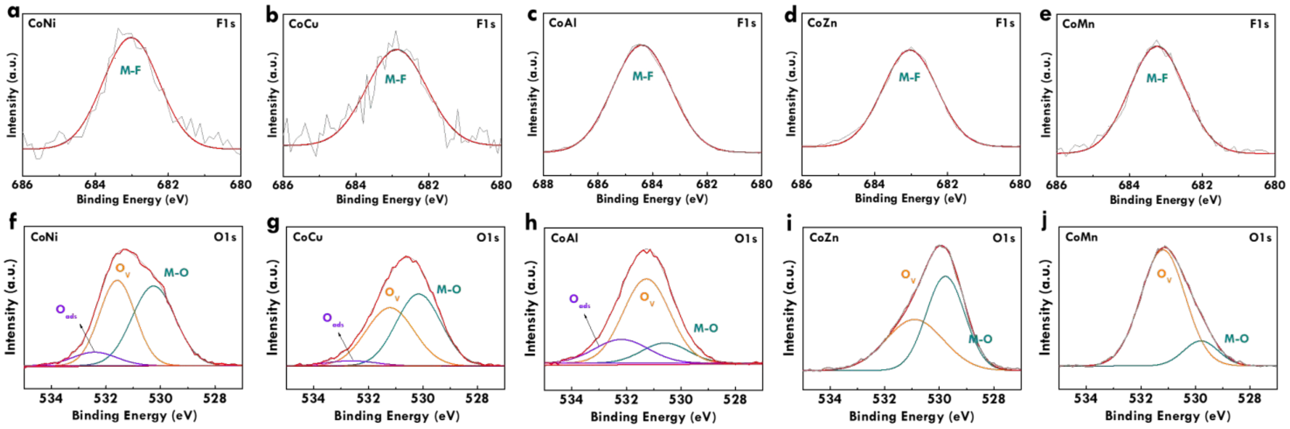

Figure S3 The F1s spectra of (a) CoNi, (b) CoCu, (c) CoAl, (d) CoZn and (e) CoMn; the O1s spectra of (f) CoNi, (g) CoCu, (h) CoAl, (i) CoZn and (j) CoMn.

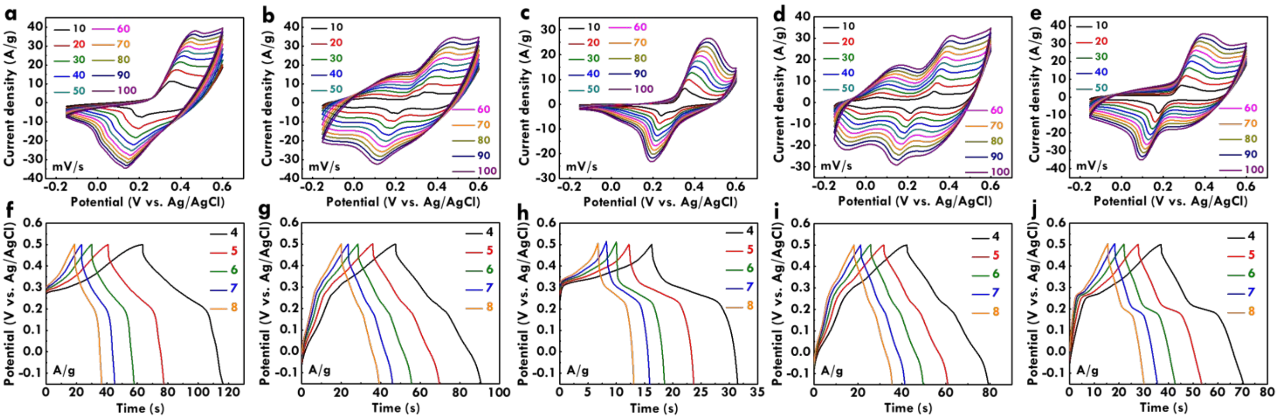

Figure S4 The CV curves at different scan rates for (a) CoNi, (b) CoCu, (c) CoAl, (d) CoZn and (e) CoMn; GC/D curves at different current densities of (f) CoNi, (g) CoCu, (h) CoAl, (i) CoZn and (j) CoMn.

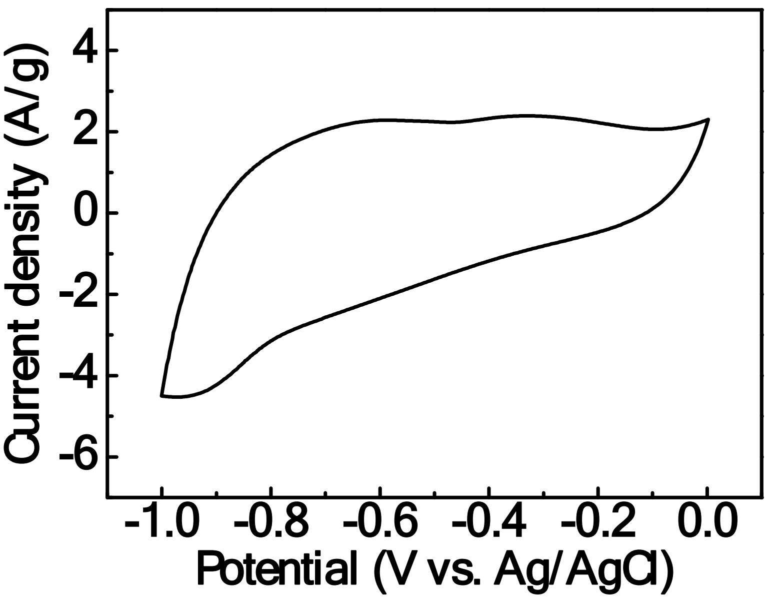

Figure S5 The CV curve of the rGO electrode.
